# Exploring Relationships Between Autism Spectrum Disorder Symptoms and Eating Disorder Symptoms in Adults With Anorexia Nervosa: A Network Approach

**DOI:** 10.3389/fpsyt.2020.00401

**Published:** 2020-05-12

**Authors:** Jess Kerr-Gaffney, Daniel Halls, Amy Harrison, Kate Tchanturia

**Affiliations:** ^1^Department of Psychological Medicine, Institute of Psychiatry, Psychology, and Neuroscience, King's College London, London, United Kingdom; ^2^Department of Psychology and Human Development, University College London, London, United Kingdom; ^3^National Eating Disorders Service, Psychological Medicine Clinical Academic Group, South London and Maudsley NHS Trust, London, United Kingdom; ^4^Department of Psychology, Ilia State University, Tbilisi, Georgia

**Keywords:** anorexia nervosa, comorbidity, autism spectrum disorder, self-report, social behavior

## Abstract

Over the past few decades, research has accumulated to suggest a relationship between anorexia nervosa (AN) and autism spectrum disorder (ASD). Elevated ASD traits are present in around one third of those with AN, and there is some evidence to suggest that ASD traits are associated with more severe eating disorder (ED) psychopathology. The current study aimed to examine relationships between ED and ASD symptoms in individuals with a lifetime history of AN using network analysis. One hundred and one participants completed the ED Examination Questionnaire (EDE-Q) and the Social Responsiveness Scale (SRS-2). A regularized partial correlation network was estimated using a graphical least absolute shrinkage and selection operator. Expected influence (EI) and bridge EI values were calculated to identify central and bridge symptoms respectively. Isolation, difficulties with relating to others, and feelings of tension during social situations were most central to the network, while poor self-confidence, concerns over eating around others, and concerns over others seeing one's body were the strongest bridge symptoms. Our findings confirm that interpersonal problems are central to ED psychopathology. They also suggest poor self-confidence and social anxiety-type worries may mediate the relationship between ED and ASD symptoms in those with a lifetime diagnosis of AN. Longitudinal studies examining fluctuations in symptoms over time may be helpful in understanding direction of causality.

## Introduction

Over the past few decades, evidence suggesting a relationship between autism spectrum disorder (ASD) and anorexia nervosa (AN) has accumulated (1, 2). ASD is a neurodevelopmental disorder characterized by difficulties in social communication and interaction, as well as restrictive, repetitive patterns of behavior or interests (3). ASD is a lifelong condition, and is more commonly diagnosed in males than females (4). On the other hand, AN is a severe eating disorder (ED) associated with persistent restriction of energy intake, fear of weight gain, and disturbances in the way in which one**'**s body shape or weight is experienced (3). AN is more prevalent in females, and peak age of onset is in late adolescence (5, 6).

Despite the apparent differences between the two disorders, empirical research has shown a number of similarities in the phenotypic expressions of AN and ASD. For example, in the socio-emotional domain, considerable research has documented difficulties in emotion recognition (7), empathy (8), and theory of mind (ToM) (9) in individuals with ASD. These difficulties are also seen in those with AN, although are often less pronounced than is seen in ASD (10–12). Furthermore, high levels of alexithymia (13, 14), social anxiety (15, 16), and differences in social attention (17–19) are associated with both disorders. In the neurocognitive domain, both AN and ASD are associated with weak central coherence (20, 21), increased attention to detail (22, 23), and difficulties in set-shifting (24, 25), an executive function that allows for flexible thinking and behavior.

As well as these similarities in socio-emotional and neurocognitive profiles, those with AN show high levels of ASD traits. For example, it is reported that between 4% and 52.5% of individuals with AN score above clinical cut-offs on diagnostic assessment tools for ASD1. It has been suggested that high levels of ASD traits found in a proportion of those with AN may be due to the effects of starvation, and do not represent true ASD (26). However, several studies have found that body mass index (BMI), which is often used as a measure of illness severity, is not associated with ASD traits in individuals with AN (27–33). A few of these studies also examined associations with illness duration, finding that those with high ASD traits had not been ill for a significantly longer period of time than those with low ASD traits (29, 33). Finally, a significant proportion of individuals recovered from AN also show elevated ASD traits compared to HCs (27, 30, 34). Therefore, it seems the association between ASD and AN is not a product of starvation, yet the exact nature of the relationship remains unclear.

However, there is some evidence to suggest that ASD symptoms are positively associated with severity of ED psychopathology in those with AN. For example, in a large sample of inpatients with AN, Tchanturia et al. (31) reported that scores on the Autism Quotient (AQ) (35) were positively associated with scores on the ED Examination Questionnaire (EDE-Q) (36). A similar association between AQ scores and ED symptoms has been reported in nonclinical populations (37, 38). Further, the presence of ASD traits in AN is associated with more frequent and longer inpatient stays (29), less improvement during treatment (39, 40), and poorer outcomes (41–43). Why might a more severe ED presentation be associated with high ASD traits? One possibility is that some of the neurocognitive traits associated with ASD, such as cognitive rigidity, increased attention to detail, and sensitivity to order may perpetuate a narrow focus on food and weight in individuals with AN and make change difficult (44). Indeed, Westwood and colleagues (45) reported that individuals with AN and high ASD traits showed higher levels of cognitive rigidity and set-shifting difficulties than individuals with low ASD traits. As well as a significant proportion of individuals with AN showing high levels of ASD traits on dimensional measures, a number of studies have found that 8%–29% meet full diagnostic criteria for ASD (34, 41, 46–49). Given that social difficulties are an important predictor of poor outcomes in AN (43, 50–52), another possibility is that those with comorbid ASD and AN have particularly poor outcomes due to the social communication difficulties associated with ASD. Yet another possibility is that avoidance of certain foods due to sensory sensitivities in ASD may reinforce food restriction. Such hypotheses remain to be tested empirically.

A potentially useful method for examining the nature of the relationship between AN and ASD symptoms is provided by network theory. Network theories of psychopathology represent psychiatric disorders as constellations of symptoms, activating one another (53). The relationships between symptoms are key to the development and maintenance of psychopathology; symptoms can form feedback loops, eventually producing a set of symptoms that are recognized as a psychiatric disorder. This theory has important implications for understanding comorbidity. Symptoms are often shared among different psychiatric disorders, for example feelings of guilt are common in obsessive compulsive disorder (OCD) and are also a central feature of major depression (3). Because symptoms in a network have causal relationships with one another, clusters of symptoms belonging to one disorder can activate those of another disorder, resulting in diagnostic comorbidity (54).

Psychological networks can be estimated using network analysis. Networks are made up of nodes (symptoms) and edges (relationships between symptoms). It is possible to calculate which nodes or symptoms have most connections in the network (node centrality) and therefore are most important in maintaining psychopathology. Further, it is possible to calculate which symptoms of a given disorder are most connected to symptoms in another disorder cluster (bridge nodes), and therefore may maintain comorbidity. Currently, only a few studies have examined comorbidity using network analysis in individuals with EDs. These studies have most often focussed on comorbidity between anxiety and ED symptoms, finding that avoidance of social eating is an important bridge symptom (55–57). Others have examined depression (55, 58) and OCD symptoms (59), however no study to date has focussed on ASD and ED symptom comorbidity.

The aim of the current study was to examine relationships between ED and ASD symptoms in individuals with AN using network analysis. Because ASD symptoms have been shown to persist in individuals recovered from AN, suggesting independence from clinical state, our sample included those with a current or past diagnosis of AN. We aimed to identify central nodes in order to understand which symptoms may be most important in maintaining the symptom network as a whole. Bridge nodes were also identified in order to detect symptoms most important in explaining potential comorbidity of AN and ASD.

## Materials and Methods

### Participants

The study was cross-sectional. Ethical approval was obtained from the National Health Service (NHS) Research Ethics Committee (Camberwell St Giles, 17/LO/1960). Participants provided their written informed consent to participate in the study. Participants with a lifetime history of AN were recruited from two specialist NHS ED services in London, online advertisements, and through the King's College London university research recruitment email. Participants were required to be between 18 and 55 years old and fluent in English. Exclusion criteria were a history of brain trauma or learning disability. A past or current diagnosis of AN was confirmed using the Structured Clinical Interview for DSM-5 Disorders, research version (SCID-5-RV) Module I “Feeding and Eating Disorders” (60).

### Procedure and Materials

Participants attended a testing session as part of a wider study on socio-emotional processing at the Institute of Psychiatry, Psychology & Neuroscience, however where participants were inpatients, testing took place at their place of treatment. Written consent was obtained, and the following measures administered:

The EDE-Q (36) was used to measure severity of ED psychopathology. Twenty-two of the 28 items are rated for frequency during the past 28 days on a seven-point Likert scale, with higher scores indicating more eating, shape, or weight concerns and behaviors. The remaining six items assessing frequency of various behaviors are not included in total or subscale score calculations, as these items can take on any value. The EDE-Q demonstrates good psychometric properties, correlating with measures of similar constructs (61). Cronbach's alpha was 0.91.

The Social Responsiveness Scale-2nd edition, adult self-report form (SRS-2) (62) is a 65-item questionnaire assessing symptoms associated with ASD, with higher scores indicating more autistic symptoms. There are five subscales: social awareness (ability to recognize social cues), social cognition (interpreting social behavior), social communication (reciprocal communication in social situations), social motivation (motivation to participate in social interactions), and restrictive interests and repetitive behavior (circumscribed interests and stereotypy). Respondents indicate their agreement with each item on a four-point Likert scale, rating their behavior over the past six months. The SRS-2 has been used extensively in ASD research, and is also recommended for use in diagnostic assessments in adults with ASD (63). Validation studies have found measurement invariance across the sexes, and few sex, age, or rater effects (64–66). Scores on the SRS-2 have been shown to predict whether individuals with AN score above the clinical cut-off on the Autism Diagnostic Observation Schedule, 2nd edition (ADOS-2) (67), a “gold-standard” clinical interview measure for ASD (68). Cronbach's alpha was 0.96.

Demographic information was also collected, along with weight and height measurements to calculate BMI (height/weight^2^).

### Network Analysis

Analyses were performed in R version 3.6.1 (69). R codes are provided in the **Supplementary Information**.

#### Item Selection

Network analysis assumes that each node in the network represents a distinct construct. Given that some of the questionnaire items are very similar in content, the goldbricker function in R package *networktools* (70) was used to select items to include in the network. Goldbricker compares dependent overlapping correlations (i.e., items with high multicollinearity) for all items in the network. If a certain proportion of correlations between node A and all other nodes do not significantly differ from those between node B and all other nodes (e.g., items share ≥75% of correlations), nodes A and B are assumed to be overlapping items measuring the same construct (“bad pairs”). One of the nodes is subsequently removed. The 22 Likert items from the EDE-Q and all 65 items from the SRS-2 were entered. After dropping the bad pairs, 18 EDE-Q and 55 SRS-2 items were left for inclusion in the network. The full list of EDE-Q and SRS-2 items and those included in the network are provided in the **Supplementary Information**.

#### Network Estimation and Accuracy

A regularized partial correlation network with weighted edges was estimated using a graphical least absolute shrinkage and selection operator (LASSO) using the *qgraph* R package (71). This method limits the total sum of absolute parameter values and drops edges that are close to zero out of the model, keeping only those that are most robust and likely to represent genuine associations. The tuning parameter (λ) was set to 0.25. This value is typically set between 0 and 0.5, with higher values resulting in simpler models with fewer edges, and lower values favoring discovery but more likely to estimate spurious edges (72).

Accuracy of edge-weights were assessed using nonparametric bootstrapping using the *bootnet* package (73). Bootstrapping involves repeatedly estimating a model under sampled or simulated data and estimating the statistic of interest, in this case, edges. The bootstrapped 95% confidence intervals (CIs) indicate the sampling variation, and the strength of a given edge is difficult to interpret if bootstrapped CIs are wide. Correlation stability (CS) coefficients were calculated to assess the stability of expected influence (EI) and bridge EI. In this case, a case-dropping bootstrap is used to indicate whether the centrality indices remain the same after reestimating the network using only a subset of cases from the sample. The CS coefficient indicates the proportion of the sample that can be dropped to retain a correlation >0.7 with the original sample. It should not be below 0.25, and preferably above 0.5 (73). Finally, bootstrapped difference tests (a = 0.05) were run to test for significant differences in centrality indices between nodes.

#### Network Interpretation

Central nodes were identified by calculating EI using the *networktools* package. EI is similar to the more commonly used metric, strength, as it is calculated by summing all of the edges a given node has with all other nodes in a network. However unlike strength, which does not distinguish between positive and negative edges, EI accounts for the direction of associations. This is an important distinction in networks which include psychopathological symptoms of more than one disorder, where some negative relationships are likely (74).

Bridge nodes were identified by calculating bridge EI using the *networktools* package. Both bridge EI one-step (bridge EI1) and bridge EI two-step (bridge EI2) were calculated. Bridge EI1 identifies the strength and directionality of the relationships a node in one cluster has with all nodes of another cluster. Bridge EI2 additionally takes into account the secondary influence of a node *via* the influences of its immediate neighbors. For centrality and bridge indices, higher values represent greater influence. Z-scores are reported throughout for ease of interpretation.

## Results

### Sample Characteristics

In total, 101 participants took part in the study. Fifty-one were acutely ill with AN, while fifty were recovered. Demographic and clinical information is displayed in **Table 1**. On the SRS-2, 43% of participants scored within the “normal” range, scores within this range are not associated with clinically significant symptoms. Seventeen percent of participants scored within the “mild” range, indicating deficiencies in reciprocal social behavior that are clinically significant and may lead to mild to moderate interference with daily living. A further 21% scored within the “moderate” range, indicating clinically significant difficulties which lead to substantial interference with social behavior. Finally, 19% of participants scored in the “severe” range, indicating severe and enduring difficulties with social behavior. Scores within the moderate and severe range are typical for individuals with a diagnosis of ASD.

**Table 1:** Demographic and clinical information of participants with lifetime anorexia nervosa (AN) (N = 101).

	Mean (SD)	Range
Age (years)	26.95 (8.27)	18.16 – 54.59
% female	95.0	–
BMI	18.39 (3.25)	12.90 – 27.00
Years of education	16.33 (2.89)	10.00 – 27.00
Illness length (years)	6.47 (6.89)	0.50 – 35.00
% on psychiatric medication	43.6	–
EDE-Q total	2.84 (1.75)	0.00 – 5.69
SRS-2 total	77.66 (33.11)	17.00 – 160.00

AN, anorexia nervosa; BMI, body mass index; EDE-Q, eating disorder examination questionnaire; SRS-2, social responsiveness scale; SD, standard deviation.

### Network Estimation and Accuracy

**Figure 1:**
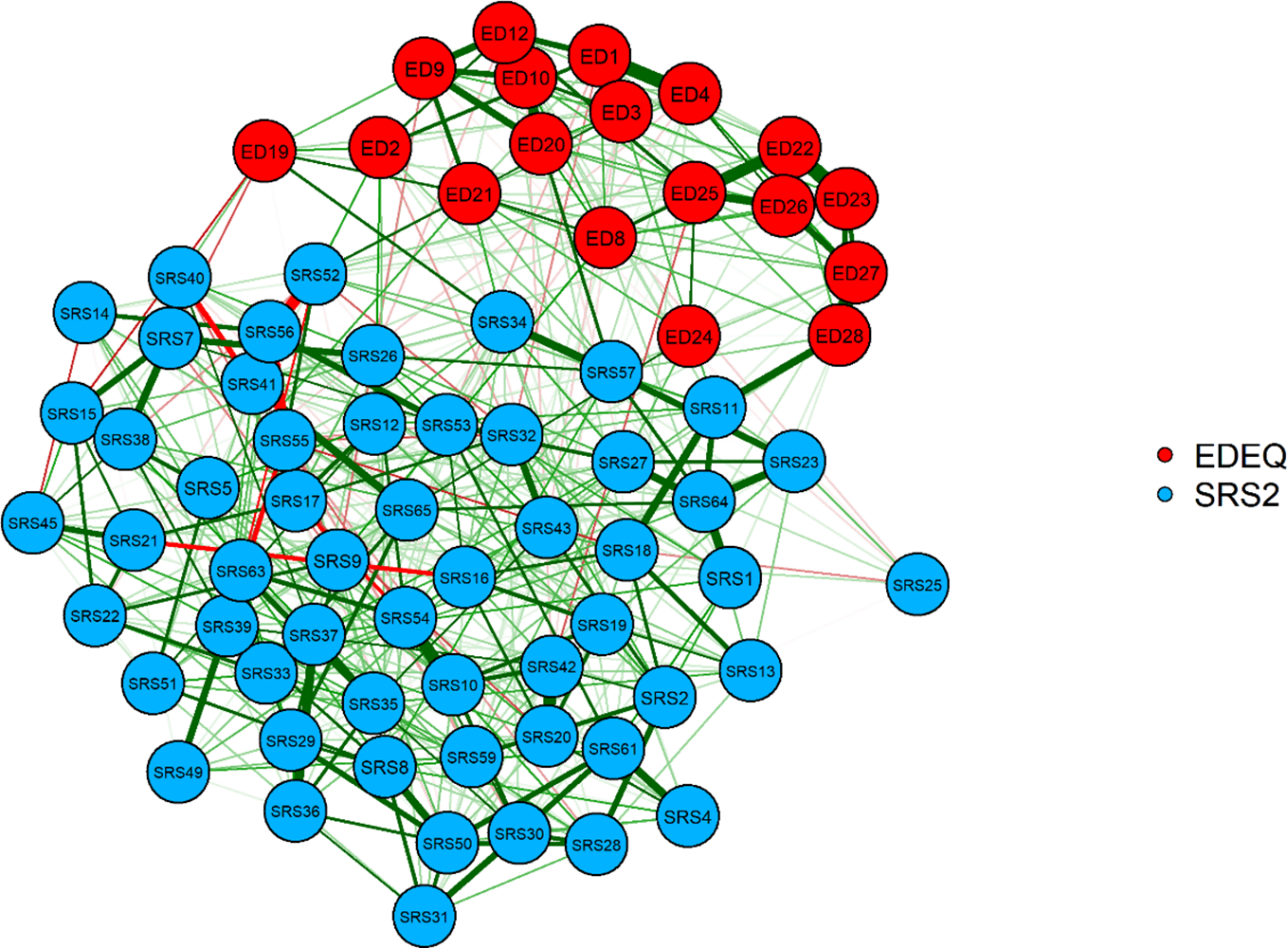
Graphical least absolute shrinkage and selection operator network. Eating Disorder Examination Questionnaire (EDE-Q) items: ED1, limit food; ED2, fasting; ED3, excluding foods; ED4, eating rules; ED8, concentration affected by shape/weight; ED9, fear of loss of control; ED10, fear of weight gain; ED12, desire to lose weight; ED19, eating in secret; ED20, guilt over eating; ED21, concern over other people seeing you eat; ED22, weight overvaluation; ED23, shape overvaluation; ED24, reaction to weighing; ED25, weight dissatisfaction; ED26, shape dissatisfaction; ED27, uncomfortable seeing own body; ED28, uncomfortable over others seeing own body. Social responsiveness scale (SRS-2) items: SRS1, uncomfortable in social situations; SRS2, facial expressions; SRS4, rigid behavior; SRS6, prefer to be alone; SRS7, aware of others feelings; SRS8, strange behavior; SRS9, dependent on others; SRS10, take things literally; SRS11, good self-confidence; SRS12, communicate feelings; SRS13, awkward in turn taking interactions; SRS14, not well coordinated; SRS15, understand change in tone/facial expression; SRS16, avoid/unusual eye contact; SRS17, recognize unfairness; SRS18, difficulty making friends; SRS19, frustrated in conversations; SRS20, sensory interests; SRS21, imitate others'; SRS22, interact appropriately; SR23, avoid social events; SRS25, don't mind being out of step with others; SR26, offer comfort to others; SRS27, avoid starting social interactions; SRS28, think about the same thing over and over; SRS29, regarded as odd; SRS30, upset in situations with lots going on; SRS31, can't get mind off something; SRS32, good personal hygiene; SRS33, socially awkward; SRS34, avoid people who want to be emotionally close to me; SRS35, have trouble keeping up with conversations; SRS36, difficulty relating to family; SRS37, difficulty relating to adults outside family; SRS38, respond to others' moods; SRS39, interested in too few topics; SRS40, imaginative; SRS41, wander aimlessly between activities; SRS42, sensory sensitivity; SRS43, enjoy small talk; SRS45, interested in what others' are attending to; SRS49, do well at intellectual tasks; SRS50, repetitive behaviors; SRS51, difficulty answering questions directly; SRS52, overly loud; SRS53, monotone voice; SRS54, thing about people and objects in the same way; SRS55, invade others' personal space; SRS56, walk between two people; SRS57, isolate myself; SRS59, suspicious; SRS61, inflexible; SRS63, unusual greeting; SRS64, tense in social settings; SRS65, stare into space.

Questionnaire data from three participants contained missing values (representing 0.08% of the total questionnaire data). Given that nodes in our network did not rely on subscale or total score calculations from questionnaires, the rest of the data from these participants was included in analyses. The network structure composed of EDE-Q and SRS-2 symptom scores is displayed in **Figure 1**. Green edges represent positive relationships, while red indicates negative ones. The thicker the edge, the stronger the regularized partial correlation.

Plots displaying the bootstrapped CIs of estimated edge-weights, bootstrapped centrality indices, and bootstrapped differences tests are reported in the **Supplementary Material**. The EI CS coefficient was 0.67, and the bridge EI CS coefficient was 0.59, indicating both EI and bridge EI can be interpreted meaningfully (73).

### Centrality

**Figure 2:**
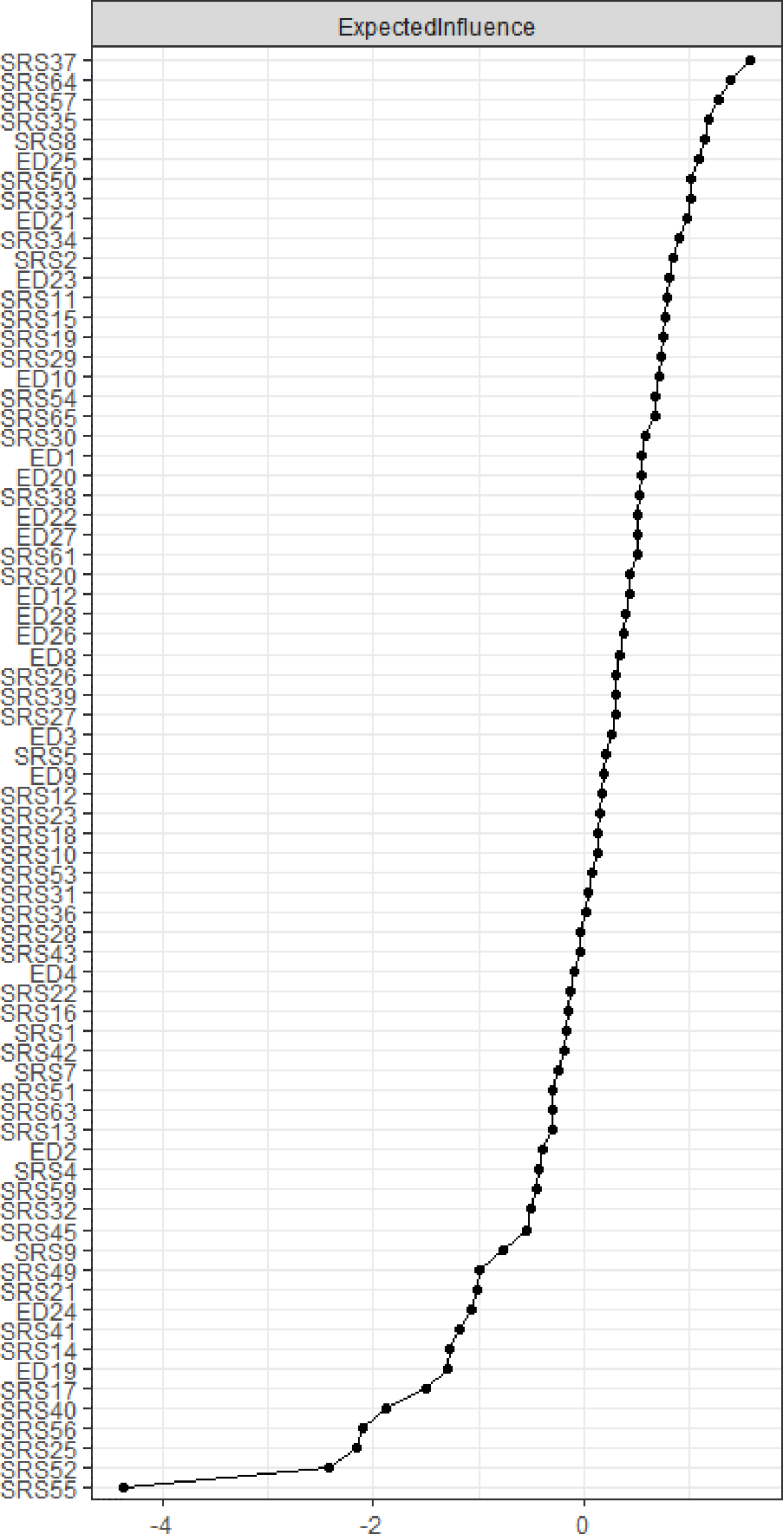
Centrality plot depicting expected influence (EI) of each node. Values are given as Z scores, higher values represent greater centrality in the network.

EI is plotted in **Figure 2**. The items with the highest EI were SRS-2 37 “I have difficulty relating to adults outside of my family,” SRS-2 57 “I tend to isolate myself,” and SRS-64 “I am much more tense in social situations than when I am by myself.”

### Bridge Nodes

**Figure 3:**
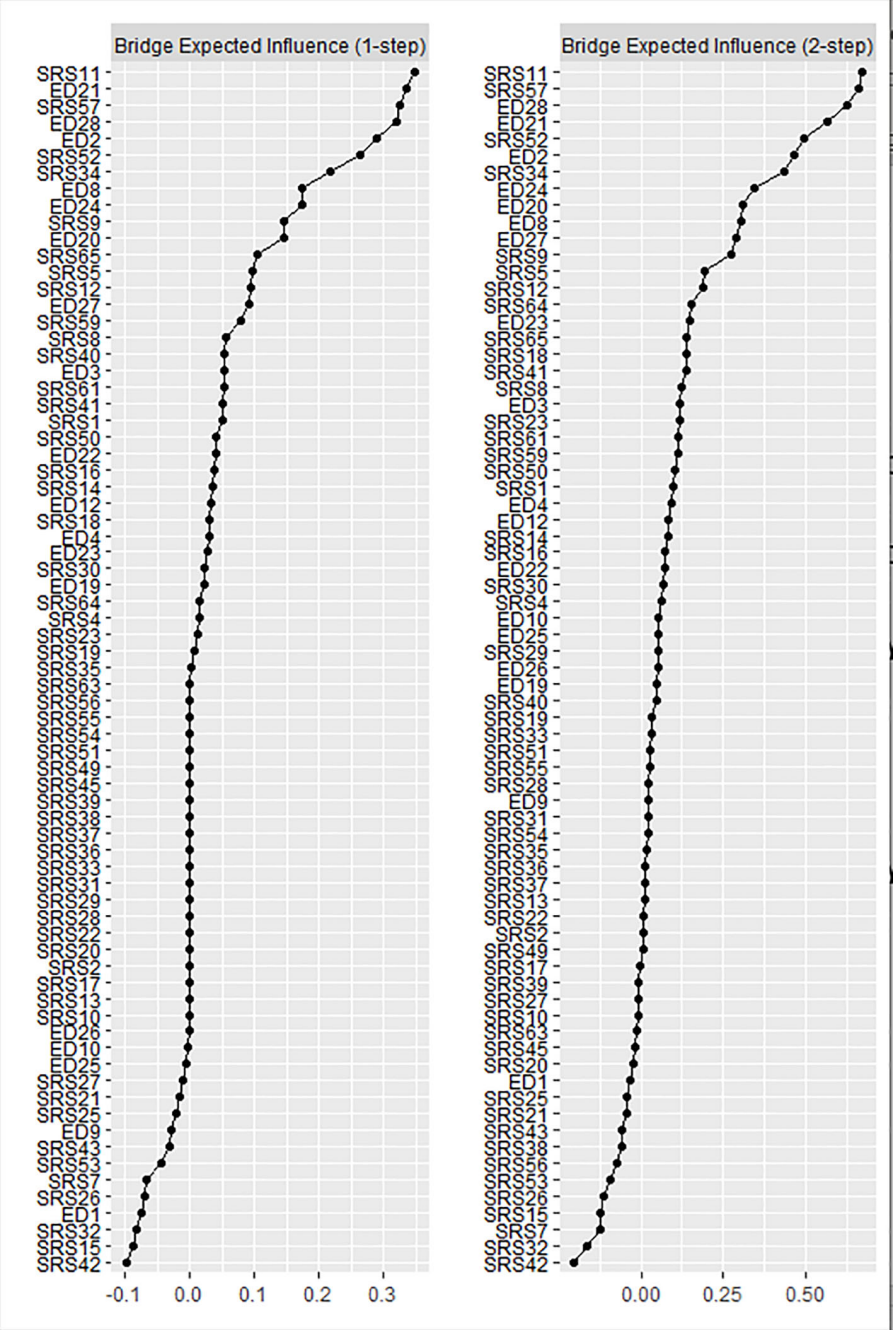
Bridge expected influence (EI) plots. Values are given as Z scores, and higher values indicate more influential nodes in the network.

Bridge EI values are plotted in **Figure 3**. For bridge EI1, the strongest ED bridge symptom was EDE-Q 21 “How concerned have you been about other people seeing you eat?”, and the strongest ASD bridge symptom was SRS-11 “I have good self-confidence” (reverse coded). For bridge EI2, the strongest ED bridge symptom was EDE-Q 28 “How uncomfortable have you felt about others seeing your shape or figure”, and the strongest ASD bridge symptom was again SRS-11 “I have good self-confidence.”

## Discussion

The current study is the first to examine relationships between ED and ASD symptoms in individuals with past or current AN using network analysis. Constructing a network of partial correlations allowed us to examine connections between symptoms, independent of the effects of other symptoms in the network. Firstly, we aimed to identify core symptoms in the network. The three nodes with the highest centrality in the network were all SRS-2 items: SRS-2 37 “I have difficulty relating to adults outside of my family,” SRS-2 57 “I tend to isolate myself”, and SRS-64 “I am much more tense in social situations than when I am by myself.” The former two items are from the social communication subscale of the SRS-2, while the latter is from the social motivation subscale. These results suggest that difficulties in social communication and isolation may be core symptoms in AN psychopathology, over and above conventional ED symptoms, such as weight and shape concern. However, it must be noted that the inclusion of recovered individuals, some of whom had rather low EDE-Q symptom scores may have influenced these results. Had our sample only included individuals in the acute stage of AN, EDE-Q items might have been more central to the network. Nonetheless, our study is not the first to demonstrate the importance of social difficulties in AN psychopathology. For example, Monteleone and colleagues (58) found that depression and personal alienation were the nodes with highest centrality in their network of symptoms in children and adolescents with AN. Personal alienation, a subscale of the EDs Inventory (EDI), reflects a sense of emotional emptiness, aloneness, and feeling separated from others. Somewhat similar findings were reported by Somli and colleagues (75), who found that in adolescents and adults with AN, depression, anxiety, interpersonal sensitivity, and ineffectiveness were most central to the network. Interpersonal sensitivity, a subscale of the Symptom Checklist 90 (SCL-90), assesses feelings of inadequacy and inferiority in comparison to others, as well as self-consciousness and discomfort during social interactions.

Our second aim was to identify bridge nodes; those that connect ED and ASD symptom clusters. The strongest ASD bridge symptom was SRS-2 11 “I have good self-confidence” (reverse coded), while the strongest ED bridge symptoms were EDE-Q 21 “How concerned have you been about other people seeing you eat?” (bridge EI1) and EDE-Q 28 “How uncomfortable have you felt about others seeing your shape or figure?” (bridge EI2). The self-confidence item belongs to the social motivation subscale of the SRS-2. Our results suggest that a lack of self-confidence may be important in understanding the link between ED psychopathology and ASD symptoms in those with lifetime AN. However, it must be noted that low self-confidence is a rather nonspecific psychiatric symptom, commonly reported in depression, anxiety, substance abuse disorders, and EDs (76). Interestingly, our finding is very similar to that of Forrest and colleagues (56), who found that the low self-confidence item of the State-Trait Anxiety Inventory (STAI) was the strongest trait anxiety bridge node linked to ED symptoms in a mixed ED sample. It could be that elevated scores on ASD assessments found in individuals with AN are partly due to high anxiety, a symptom shared by both disorders. In an analysis of 18 previously published comorbidity networks, Jones and colleagues (54) observed that several symptoms emerged as bridge symptoms across multiple networks. The networks included several different disorders, including anxiety, bulimia nervosa (BN), OCD, depression, and ASD, and also used a wide variety of symptom scales. This demonstrates that certain symptoms may not only explain comorbidity between two disorders, but may be important transdiagnostic factors across psychiatric disorders.

Regarding ED bridge nodes, it is notable that both involve concern over being observed by others. The concern over others seeing you eat item has repeatedly been shown to be the strongest ED bridge node connecting ED and anxiety symptoms in those with AN (55) and mixed ED groups (56, 57). High social anxiety is a feature of both ASD (16, 77) and AN (15), and our findings might suggest that social anxiety worries are important in explaining comorbidity between AN and ASD. It has been hypothesized that some of the core symptoms of ASD may increase the risk of developing social anxiety (78, 79). For example, poor social skills or difficulties in recognizing emotions and mental states in others may lead to rejection from peers and isolation during formative years, factors which are implicated in the development of social anxiety disorder (SAD). There is also evidence to suggest that SAD may be a risk factor for the development of AN. In those with both disorders, SAD precedes AN onset in around two thirds of cases (80, 81). Fears around eating in front of others may lead to avoidance of social eating in those with SAD, a potential pathway by which other eating disordered behaviors may form. Qualitative work has also provided some insight into how these factors may interact and contribute to the development of AN. For example, Kinnaird et al. (82) found that participants with AN and ASD felt that their ED had developed as a way of dealing with the social confusion and difficulties relating to other people associated with their ASD. Participants also described how social difficulties, such as dealing with noise and social chat during meal times, made inpatient treatment difficult. Similarly, based on their qualitative study of AN and ASD comorbidity, Brede and colleagues (83) hypothesized that AN may develop through both direct and indirect pathways. In the indirect pathway, ASD-related difficulties are proposed to give rise to negative emotional consequences, and restrictive eating behaviors are employed as an attempt to cope with this. For example, individuals with ASD may have a longstanding history of being bullied and socially ostracized, resulting in low self-esteem and emotional distress. Restricting food intake can provide a sense of control and numbing of strong emotions. Given our study was cross-sectional, hypotheses about the direction of causality between symptoms are preliminary. Future longitudinal research using network analysis could help disentangle interactions between ASD, ED, and social anxiety symptoms.

Our findings have important theoretical and clinical implications. That the symptoms most central to the network all concerned social difficulties provides support for models emphasizing the role of interpersonal problems in the development and maintenance of AN. For example, the cognitive-interpersonal maintenance model of AN proposes that anxious, avoidant, and socio-emotional traits, including sensitivity to stress and negative emotions, anxious and avoidant attachment, and negative self-evaluations are predisposing factors (44). While some interpersonal difficulties are worsened by the ill state, this group of traits are proposed to be present before and after the illness, and often also in family members. Further, our results support the need for therapies to target interpersonal functioning as a key maintaining factor in individuals with AN, such as interpersonal psychotherapy (84), cognitive behavioral therapy (CBT) (85), and the Maudsley model of AN treatment for adults (MANTRA) (86). Secondly, it is of note that none of the items from the restrictive interests and repetitive behavior subscale of the SRS-2 had particularly high EI or bridge EI values. This may suggest that unlike social and communication difficulties, this group of symptoms play a relatively small part in AN and ASD comorbidity. Indeed, previous studies have shown that while social and communication difficulties are often elevated in those with AN, restricted and repetitive behaviors are often less pronounced (33, 49). This might be in part due to AN samples being mostly female, a factor which is associated with lower levels of restricted interests in those with ASD (87). However, there is some evidence to suggest that males and females with ASD show differences in the types of topics they are interested in (e.g., people/animals in females rather than objects/things in males), therefore ASD symptom rating scales may not be sensitive to more female-typical presentations (88) Finally, our study examined relationships between symptoms on a group basis, however an interesting direction for future research would be to construct networks based on an individual basis. It is now possible to examine temporal associations between symptoms within individuals, potentially providing insight into which symptoms may be maintaining psychopathology and therefore could be targeted during treatment (89). Thus, network analysis could be a useful tool in the move toward more personalized treatments in psychiatry.

Several limitations of the current study should be noted. Our sample size was relatively small given the number of items included in the network, therefore the findings require replications in larger samples. Nonetheless, our stability analyses indicated the centrality indices were stable enough to be interpreted meaningfully. Secondly, only items from self-report questionnaires were considered as nodes in the network. It is likely that vulnerability factors not measured in this study are also important in explaining comorbidity between AN and ASD. For example, given the similarities in neuropsychological profiles, performance on set-shifting or other tests of executive functioning could be included as nodes in comorbidity networks. Although other aspects of psychopathology and social cognition were collected as part of our wider study, these were not included as we wanted to focus on ASD and AN comorbidity specifically, and adding more nodes to the network may have resulted in reductions in the stability and accuracy of the network. Finally, although we confirmed a past or current diagnosis of AN in our sample, we did not confirm whether participants held a diagnosis of ASD. Although scores on the SRS-2 suggested high levels of ASD traits in our sample, there may be qualitative differences in relations between symptoms between individuals with lifetime AN who do and do not have a formal diagnosis of ASD. Previous research suggests around 10% of those with AN meet full diagnostic criteria, and a further 40% display high ASD traits (49). Future studies using a network analytic approach may be useful in establishing which symptoms reflect “true” ASD, and which may be a consequence of starvation.

In conclusion, our results suggest that isolation, difficulties in relating to others, and feelings of tension during social situations may be central symptoms maintaining AN and ASD psychopathology. These symptoms are most strongly connected to other symptoms in the network, and it is suggested that targeting these symptoms in treatment may lead to improvements in the mental health of individuals with past or current AN who also show ASD traits. It must be noted that while central symptoms may be causally influential, longitudinal studies are required to confirm the directionality of relationships between symptoms. We also identified bridge nodes from each disorder cluster; those with the strongest connections to symptoms to the other symptom cluster. Poor self-confidence (ASD cluster), concern around social eating (ED cluster), and concern over other's seeing one's body were (ED cluster) were the strongest bridge symptoms. These symptoms may be important in understanding AN and ASD comorbidity.

## Data Availability Statement

The data that support the findings of this study are available on request from the corresponding author. The data are not publicly available due to privacy or ethical restrictions.

## Ethics Statement

The studies involving human participants were reviewed and approved by NHS Camberwell St Giles Research Ethics Committee. The patients/participants provided their written informed consent to participate in this study.

## Author Contributions

JK-G contributed to the conception and design of the study. JK-G and DH performed the statistical analysis. JK-G wrote the manuscript. DH, AH, and KT contributed to manuscript revision, read and approved the submitted version. KT lead the research group under which the study took place.

## Funding

JK-G is supported by a doctoral studentship from the Economic and Social Research Council (ESRC), and received research funding from the Psychiatry Research Trust. AH is funded by the Medical Research Council (MRC) (MR/S020381/1). KT would like to acknowledge: MRC-MRF fund (MR/R004595/1); the Health Foundation, an independent charity committed to bring better health care for people in the UK (1115447); and the Maudsley Charity for their support.

## Conflict of Interest

The authors declare that the research was conducted in the absence of any commercial or financial relationships that could be construed as a potential conflict of interest.
