# Supplementary material for: Exploring Relationships Between Autism Spectrum Disorder Symptoms and Eating Disorder Symptoms in Adults With Anorexia Nervosa: A Network Approach
Source: Front Psychiatry. 2020 May 12;11:401. doi: 10.3389/fpsyt.2020.00401 (PMC7235355; doi:10.3389/fpsyt.2020.00401)
Supplement: Supplementary file 1 [file DataSheet_1.docx]

Supplementary Material

# R codes

# retrieve data file and load library

Data <- read.csv("edeSRS.csv")[-1]

library(qgraph)

library(networktools)

library(bootnet)

View(Data)

#goldbricker

gb_edsrs <- goldbricker(Data, p = 0.05, threshold = 0.25)

#drop bad pairs

reduced_edsrs <- net_reduce(data = Data, badpairs = gb_edsrs, method="best_goldbricker")

#or combine pairs

reduced_edsrs <- net_reduce(data = Data, badpairs = gb_edsrs)

#save reduced data set

save(reduced_edsrs, file = "reduced_edrs.Rdata")

write.csv(reduced_edsrs, file = "reduced_edrs.csv", row.names=F)

#import reduced data set

reduced_edsrs <- read.csv("reduced_edrs.csv")

#create groups

groups <- list(EDEQ = c(1:18), SRS2 = c(19:73))

#create correlation matrix

cor <-cor_auto(reduced_edsrs)

#create EBIC graph

EBICgraph <-qgraph(cor, graph = "glasso", layout = "spring", labels = colnames(reduced_edsrs), tuning = 0.25, sampleSize = nrow(reduced_edsrs), groups = groups)

#save EBIC graph

EBICgraph <-qgraph(cor, graph = "glasso", layout = "spring", labels = colnames(reduced_edsrs), tuning = 0.25, sampleSize = nrow(reduced_edsrs), groups = groups, filetype='png')

#plot expected influence centrality

centralityPlot(EBICgraph, include = "ExpectedInfluence", orderBy = "ExpectedInfluence")

#plot bridge metrics

b <- bridge(EBICgraph, communities = groups)

plot(b, order = "value", include = c("Bridge Expected Influence (1-step)", "Bridge Expected Influence (2-step)"))

#Estimates network for bootstrapping

Network_reduced <- estimateNetwork(reduced_edsrs, default = 'cor')

#Bootstraps, nonparametric

Results_bridge_ei_nonparameteric <- bootnet(Network_reduced, default='EBICglasso', type='nonparametric',

nBoots = 1000, nCores = 8, statistics = c('bridgeExpectedInfluence', 'edge', 'strength', 'expectedInfluence'))

# Plot bootstrapped edge CIs:

plot(Results_bridge_ei_nonparameteric, labels = TRUE, order = 'sample',statistics = 'edge')

# Plot significant differences (alpha = 0.05) of edges, expected influence, expected bridge influence :

plot(Results_bridge_ei_nonparameteric, "edge", plot = "difference",onlyNonZero = TRUE,

order = "sample")

plot(Results_bridge_ei_nonparameteric, "expectedInfluence", plot = "difference")

plot(Results_bridge_ei_nonparameteric, " 'bridgeExpectedInfluence'", plot = "difference")

# Case-drop bootstrap. This is needed to compute cs-coefficients and stability

Results_case_bridge_ei <- bootnet(Network_reduced, nBoots = 2000, nCores = 10,

type = "case", default='EBICglasso', statistics = c('bridgeExpectedInfluence', 'edge', 'strength', 'expectedInfluence'))

# Plot centrality stability:

plot(Results_case_bridge_ei, statistics = c('bridgeExpectedInfluence','expectedInfluence',))

# Compute CS-coefficients:

corStability(Results_case_bridge_ei)

| **Supplementary Table 1**. Scale items | |
| --- | --- |
| Eating Disorder Examination Questionnaire (EDE-Q) | 1. Have you been deliberately trying to limit the amount of food you eat to influence your shape or weight (whether or not you have succeeded)?  2. Have you gone for long periods of time (8 waking hours or more) without eating anything at all in order to influence your shape or weight?  3. Have you tried to exclude from your diet any foods that you like in order to influence your shape or weight (whether or not you have succeeded)?  4. Have you tried to follow definite rules regarding your eating (for example, a calorie limit) in order to influence your shape or weight (whether or not you have succeeded?  5. Have you had a definite desire to have any empty stomach with the aim of influencing your shape or weight?†  6. Have you had a definite desire to have a totally flat stomach?†  7. Has thinking about food, eating, or calories made it very difficult to concentrate on things you are interested in (for example, working, following a conversation, or reading)?†  8. Has thinking about shape or weight made it very difficult to concentrate on things you are interested in (for example, working, following a conversation, or reading)?  9. Have you had a definite fear of losing control over eating?  10. Have you had a definite fear that you might gain weight?  11. Have you felt fat?†  12. Have you had a strong desire to lose weight?  13. Over the past 28 days, how many times have you eaten what other  people would regard as an unusually large amount of food (given the  circumstances)? ††  14. …On how many of these times did you have a sense of having lost  control over your eating (at the time that you were eating)? ††  15. Over the past 28 days, how many DAYS have such episodes of  overeating occurred (i.e., you have eaten an unusually large amount of  food and have had a sense of loss of control at the time)? ††  16. Over the past 28 days, how many times have you made yourself sick  (vomit) as a means of controlling your shape or weight? ††  17. Over the past 28 days, how many times have you taken laxatives as a  means of controlling your shape or weight? ††  18. Over the past 28 days, how many times have you exercised in a  “driven” or “compulsive” way as a means of controlling your weight,  shape, or amount of fat, or to burn off calories? ††  19. Over the past 28 days, on how many days have you eaten in secret (i.e., furtively)?  20. On what proportion of the times that you have eaten have you felt guilty (felt that you’ve done wrong) because of its effect on your shape or weight?  21. Over the past 28 days, how concerned have you been about other people seeing you eat?  22. Has your weight influenced how you think about (judge) yourself as a person?  23. Has your shape influenced how you think about (judge) yourself as a person?  24. How much would it have upset you if you had been asked to weigh yourself once a week (no more, or less, often) for the next four weeks?  25. How dissatisfied have you been with your weight?  26. How dissatisfied have you been with your shape?  27. How uncomfortable have you felt seeing your body (for example, seeing your shape in the mirror, in a shop window reflection, while undressing or taking a bath or shower)?  28. How uncomfortable have you felt about others seeing your shape or figure (for example, in communal changing rooms, when swimming, or wearing tight clothes? |
| Social Responsiveness Scale, adult self-report version (SRS-2) | 1. I am much more uncomfortable in social situations than when I am by myself.  2. My facial expressions send the wrong message to others about how I actually feel.  3. I feel self-confident when interacting with others.†  4. When under stress, I engage in rigid or inflexible patterns of behaviour that seem odd to people.  5. I do not recognize when others are trying to take advantage of me.†  6. I would rather be alone than with others.  7. I am usually aware of how others are feeling.  8. I behave in ways that seem strange or bizarre to others.  9. I am overly dependent on others for help with meeting my everyday needs.  10. I take things too literally, and because of that, I misinterpret the intended meaning of parts of a conversation.  11. I have good self-confidence.  12. I am able to communicate my feelings to others.  13. I am awkward in turn taking interactions with others (for example, I have a hard time keeping up with the give-and-take of a conversation.  14. I am not well coordinated.  15. When people change their tone or facial expression, I usually pick up on that and understand what it means.  16. I avoid eye contact or am told that I have unusual eye contact.  17. I recognise when something is unfair.  18. I have difficulty making friends, even when I’m trying my best.  19. I get frustrated trying to get ideas across in conversations.  20. I have sensory interests that others find unusual (for example, smelling or looking at things in a special way).  21. I am able to imitate others’ actions and expressions when it is socially acceptable to do so.  22. I interact appropriately with other adults.  23. I do not join group activities or social events unless prompted or strongly urged to do so.  24. I have more difficulty than others with changes in my routine.†  25. I do not mind being out of step with or “not on the same wavelength” as others.  26. I offer comfort to others when they are sad.  27. I avoid starting social interactions with other adults.  28. I think or talk about the same thing over and over.  29. I am regarded by others as odd or weird.  30. I become upset in situations with lots of things going on.  31. I can’t get my mind off something once I started thinking about it.  32. I have good personal hygiene.  33. My behaviour is socially awkward, even when I’m trying to be polite.  34. I avoid people who want to be emotionally close to me.  35. I have trouble keeping up with the flow of normal conversation.  36. I have difficulty relating to family members.  37. I have difficulty relating to adults outside of my family.  38. I respond appropriately to mood changes in others (for example, when a friend’s mood changes from happy to sad).  39. People think I am interested in too few topics, or that I get carried away with those topics.  40. I am imaginative.  41. I sometimes seem to wander aimlessly from one activity to another.  42. I am overly sensitive to certain sounds, textures, or smells.  43. I enjoy small talk (casual conversation with others).  44. I have more trouble than most people with understanding chains of causation (in other words, how events are related to one another).†  45. When others around me are paying attention to something, I get interested in what they are attending to.  46. Others feel that I have overly serious facial expressions.†  47. I laugh at inappropriate times.†  48. I have a good sense of humor and can understand jokes.†  49. I do extremely well at certain kinds of intellectual tasks, but do not do as well at most other tasks.  50. I have repetitive behaviours that others consider odd.  51. I have difficulty answering questions directly and end up talking around the subject.  52. I get overly loud without realizing it.  53. I tend to talk in a monotone voice (in other words, less inflection of voice than most people demonstrate).  54. I tend to think about people in the same way I do objects.  55. I get too close to others of invade their personal space without realizing it.  56. I sometimes make the mistake of walking between two people who are trying to talk to one another.  57. I tend to isolate myself.  58. I concentrate too much on parts of things rather than seeing the whole picture.†  59. I am more suspicious than most people.  60. Other people think I am emotionally distant and do not show my feelings.†  61. I tend to be inflexible.  62. When I tell someone my reason for doing something, it strikes the person as unusual or illogical.†  63. My way of greeting another person is unusual.  64. I am much more tense in social settings than when I am by myself.  65. I find myself staring or gazing off into space. |
| †Item was removed by goldbricker  ††Frequency item not included | |


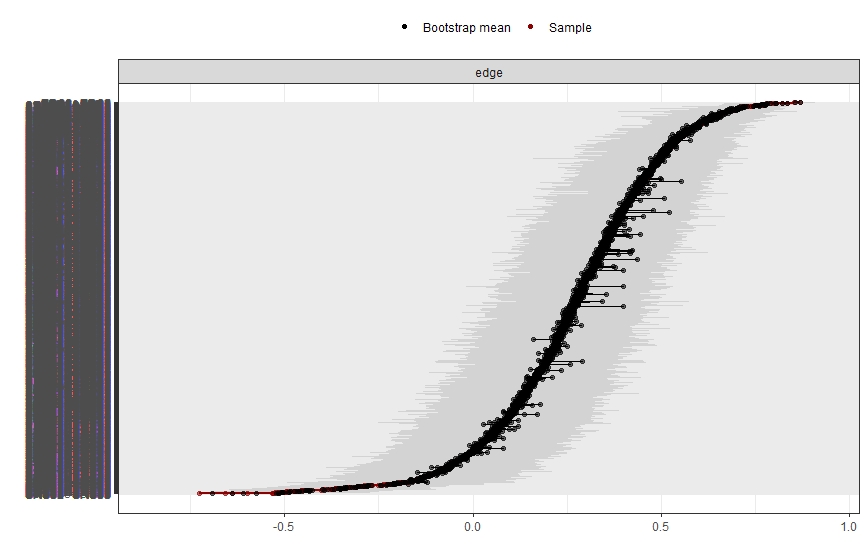


**Supplementary Figure 1.** Bootstrapped confidence intervals (CIs, grey areas) of estimated edge weights. Each horizontal line represents an edge in the network, ordered from the edge with the highest weight to the edge with weight. y-axis labels have been removed to avoid cluttering.


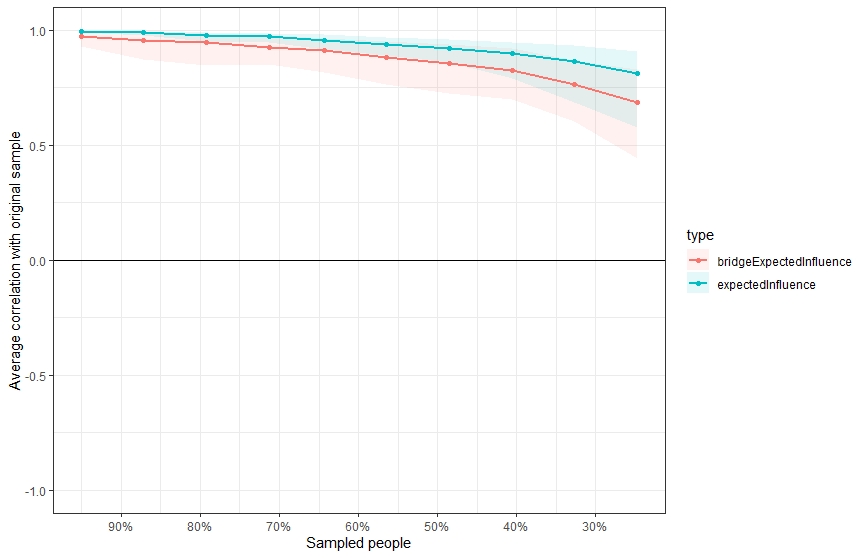


**Supplementary Figure 2.** Bootstrapped expected influence and bridge expected influence stability. Solid lines indicate centrality correlation coefficients, shaded areas are 95% confidence intervals.


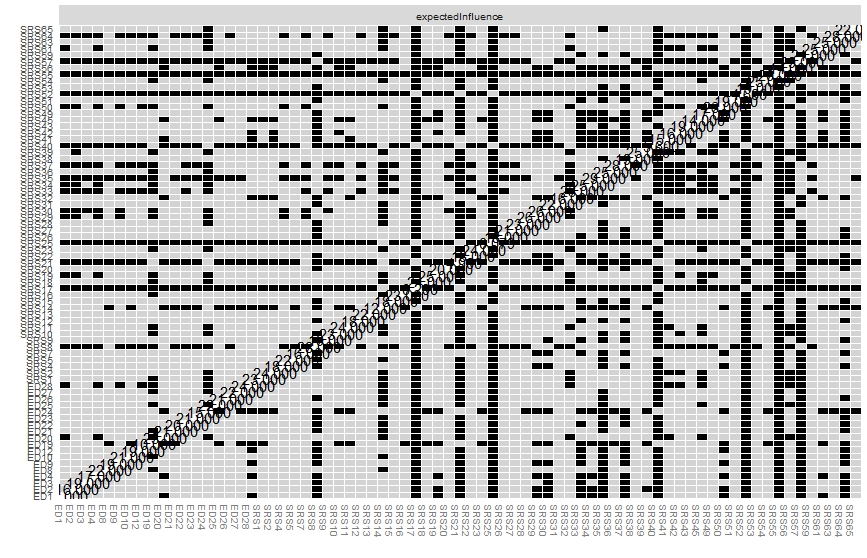


**Supplementary Figure 3.** Bootstrapped difference tests between symptoms in the network. Black boxes indicate a significant difference in expected influence centrality between two symptoms, grey boxes represent nonsignificant differences.


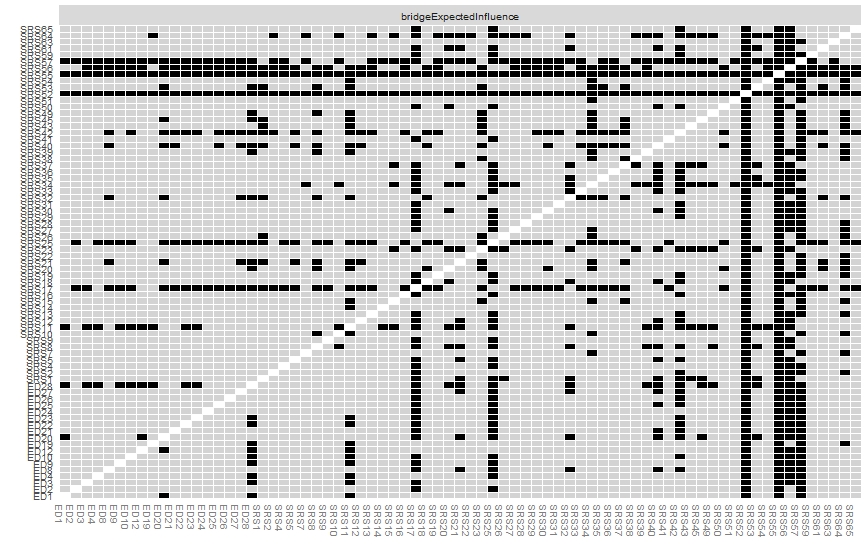


**Supplementary Figure 4.** Bootstrapped difference tests between symptoms in the network. Black boxes indicate a significant difference in bridge expected influence between two symptoms, grey boxes represent nonsignificant differences.
